# REASSESSING POSITIVE RESULTS: DETECTION BIAS IN EPIDEMIOLOGIC DEMENTIA STUDIES OF ELECTRONIC HEALTH RECORD DATA

**DOI:** 10.1093/geroni/igae098.0530

**Published:** 2024-12-31

**Authors:** Jingxuan Wang, Minhyuk Choi, Scott Zimmerman, Ruijia Chen, Cathy Schaefer, Deborah Blacker, M Maria Glymour

**Affiliations:** University of California San Francisco, San Francisco, California, United States; University of California San Francisco, San Francisco, California, United States; University of California San Francisco, San Francisco, California, United States; Boston University, Lexington, Massachusetts, United States; Kaiser Permanente Northern California, Oakland, California, United States; Massachusetts General Hospital, Boston, Massachusetts, United States; Boston University, Boston, Massachusetts, United States

## Abstract

Many epidemiologic studies of dementia use electronic health record (EHR) data, which are vulnerable to detection bias. Dementia is more likely to be detected when patients have more contact with the healthcare system. Using EHR data from the UK Biobank (UKB) and All of Us (AoU), we evaluated potential detection bias by estimating associations of three negative control exposures with dementia. We included 298,784 UKB participants and 105,836 AoU participants aged ≥ 55 without baseline dementia and estimated associations of kidney stone, forearm fracture, and urinary tract infection (UTI) with incident dementia. All diagnoses were ascertained from EHR data. We used Cox models to evaluate associations of binary exposure with all-cause dementia overall and in 4 time intervals after the exposure (0–1, >1–5, >5–10, and >10 years) compared to individuals with no prior exposure. All models adjusted for demographic and socioeconomic factors. Our results showed that all three exposures were associated with an elevated risk of dementia, especially in the first year after exposure diagnosis. The association attenuated in subsequent years. It suggests the chance of receiving a dementia diagnosis increases sharply in the first year following a diagnosis of kidney stone, forearm fracture, or UTI, although the influence of these conditions on dementia pathology is likely to be small or null. These short-term increases likely represent detection bias, i.e., they would not have received a dementia diagnosis at that time in the absence of the medical attention necessitated by kidney stone, forearm fracture or UTI.

